# Biodegradable Hydrogel Scaffolds Based on 2-Hydroxyethyl Methacrylate, Gelatin, Poly(β-amino esters), and Hydroxyapatite

**DOI:** 10.3390/polym14010018

**Published:** 2021-12-22

**Authors:** Vuk V. Filipović, Marija M. Babić Radić, Jovana S. Vuković, Marija Vukomanović, Marina Rubert, Sandra Hofmann, Ralph Müller, Simonida Lj. Tomić

**Affiliations:** 1 University of Belgrade, Institute of Chemistry, Technology and Metallurgy, Njegoseva 12, 11000 Belgrade, Serbia; vukan87@yahoo.com; 2 University of Belgrade, Faculty of Technology and Metallurgy, Karnegijeva 4, 11000 Belgrade, Serbia; mbabic@tmf.bg.ac.rs (M.M.B.R.); jjovasevic@tmf.bg.ac.rs (J.S.V.); 3 Advanced Materials Department, Jožef Stefan Institute, Jamova Cesta 39, 1000 Ljubljana, Slovenia; marija.vukomanovic@ijs.si; 4 Institute for Biomechanics, ETH Zurich, Leopold-Ruzicka-Weg 4, 8093 Zurich, Switzerland; marinarubert@gmail.com (M.R.); s.hofmann@tue.nl (S.H.); ram@ethz.ch (R.M.); 5 Department of Biomedical Engineering, Institute for Complex Molecular Systems, Eindhoven University of Technology, 5600 Eindhoven, The Netherlands

**Keywords:** 2-hydroxyethyl methacrylate/PBAE/gelatin/dopped hydroxyapatite, hydrogel scaffolding biomaterial, biodegradable scaffolds, biocompatibility, tissue regeneration engineering

## Abstract

New composite 3D scaffolds were developed as a combination of synthetic polymer, poly(2-hydroxyethyl methacrylate) (PHEMA), and a natural polymer, gelatin, with a ceramic component, nanohydroxyapatite (ID nHAp) dopped with metal ions. The combination of a synthetic polymer, to be able to tune the structure and the physicochemical and mechanical properties, and a natural polymer, to ensure the specific biological functions of the scaffold, with inorganic filler was applied. The goal was to make a new material with superior properties for applications in the biomedical field which mimics as closely as possible the native bone extracellular matrix (ECM). Biodegradable PHEMA hydrogel was obtained by crosslinking HEMA by poly(β-amino esters) (PBAE). The scaffold’s physicochemical and mechanical properties, in vitro degradation, and biological activity were assessed so to study the effects of the incorporation of nHAp in the (PHEMA/PBAE/gelatin) hydrogel, as well as the effect of the different pore-forming methods. Cryogels had higher elasticity, swelling, porosity, and percent of mass loss during degradation than the samples obtained by porogenation. The composite scaffolds had a higher mechanical strength, 10.14 MPa for the porogenated samples and 5.87 MPa for the cryogels, but a slightly lower degree of swelling, percent of mass loss, and porosity than the hybrid ones. All the scaffolds were nontoxic and had a high cell adhesion rate, which was 15–20% higher in the composite samples. Cell metabolic activity after 2 and 7 days of culture was higher in the composites, although not statistically different. After 28 days, cell metabolic activity was similar in all scaffolds and the TCP control. No effect of integrating nHAp into the scaffolds on osteogenic cell differentiation could be observed. Synergetic effects occurred which influenced the mechanical behavior, structure, physicochemical properties, and interactions with biological species.

## 1. Introduction

Great progress in the field of tissue engineering (TE) was accomplished in the past few decades due to the multidisciplinary research involving cell biology, biomaterials design and processing, imaging, surface characterization, and functionalization for improved cell–material interactions [1]. Hydrogel three-dimensional scaffolds play a key role in TE and regenerative medicine as biocompatible, biodegradable, and noncytotoxic, nonimmunogenic materials which can provide mechanical, physical, and chemical support for cell adhesion, proliferation, and differentiation in vitro and in vivo, leading to the regeneration of the affected tissue [2]. Polymeric hydrogels provide porous, highly hydrophilic 3D structures. Their high water content ensures a soft texture similar to native tissue, which can mimic some of the physical and chemical properties of the natural ECM [3]. Such hydrogels are beneficial for cell survival and support the growth of the new tissue and, additionally, they can reduce the inflammatory response of the body [4,5]. Hydrogels also possess the capacity to encapsulate cells and bioactive molecules and to degrade once their task of forming new tissue is accomplished and are thus distinguished as superior biomaterials for 3D scaffolding biomaterials [6].

Various polymers, including natural, synthetic, and natural/synthetic hybrids, have been used to make hydrogel 3D scaffolds for use in tissue engineering via chemical or physical crosslinking. The basic limitation of synthetic hydrogels is a lack of cell-specific bioactivities and, for most of them, the ability to degrade. Natural polymers are biocompatible, biodegradable, and have better biological activity but poor mechanical properties, potential immunogenic reactions, and batch-to-batch variability significantly limit their use in TE [7]. One of the possibilities to overcome the limitations of synthetic and natural hydrogels is to make hybrid structures, as a combination of natural and synthetic polymers, securing the tunable physical properties, and natural polymer, providing the specific biological functions [8,9]. Many naturally occurring biopolymers, such as collagen, gelatin, fibrinogen, hyaluronic acid, chitosan, and heparin, have been used to make hybrid hydrogels with synthetic polymers, such as poly(ethylene glycol) (PEG)**,** poly(*N*-isopropyl acrylamide) (PNIPAm), and poly(vinyl alcohol) (PVA) [10]. PHEMA was very rarely used in such hybrid polymeric materials due to its nonbiodegradability. Another improvement of the mechanical and biological characteristics of hydrogel scaffolds for use in bone tissue engineering can be accomplished by introducing biopolymer/bioceramic nanocomposites [11]. Hydroxyapatite (Ca_10_(PO_4_)_6_(OH)_2_, HAp) constitutes the largest portion of inorganic components in human bones. It is a typical bioceramic clinically used as a bone substitute and which is also used in bone tissue engineering [12] to improve bone cement mechanical properties. Hydroxyapatite can increase the concentration of local Ca^2+^, which can activate the proliferation of osteoblasts and promote the growth and differentiation of mesenchymal stem cells (MSC) [13]. Hydroxyapatite mineralization of PHEMA has been studied by several authors [14,15]. In the case of tissue regeneration, the surface of the nHAp particles sorbs proteins serving as inductors of cell origin. Recently, ion-doped nHAp (ID nHAp) has shown attractive properties in load-bearing bone tissue enhanced osteoconductivity compared with hydroxyapatite [16].

A typical composite material in the human body is bone, representing a combination of organic components, collagen, and inorganic hydroxyapatite, with specific properties essential for bone functions. Materials used today in bone tissue engineering and regeneration tend to be as similar as possible to those found in nature. Therefore, synthetic and natural polymers are often combined with inorganic materials to obtain biocompatible and biodegradable composite materials. Tunable structure, physicochemical and mechanical properties, and biological activity are desirable in such composites to enable the closest resemblance to the human bone. In large fractures that would not heal by themselves, composite polymeric biomaterials are needed in combination with bone as synthetic bone-graft substitutes.

In our previous work, gelatin and 2-hydroxyethyl methacrylate (HEMA) crosslinked with poly(β-amino ester) (PBAE) macromers were used for the first time to prepare biodegradable hybrid 3D scaffolds for TE applications, as an interpenetrating polymer network (IPN) structure of a natural and synthetic polymer [17]. Since PHEMA has excellent biocompatibility, cytocompatibility, and tunable mechanical properties, as well as a minimal immunological response, it was used in this work. PHEMA biodegradability was provided by crosslinking HEMA with degradable PBAE crosslinkers. The study indicated that HEMA/gelatin/PBAE scaffolds represent a new class of polymeric biomaterials with favorable functional properties, swelling, and mechanical characteristics, along with slow degradation rates, which can be tuned by changing the PBAE crosslinker composition or molecular weight, for the potential application as scaffolding biomaterials in tissue regeneration. The novelty in the present work, concerning the above mentioned one, was that biodegradable PHEMA and gelatin hydrogels were combined with a ceramic component, nanohydroxyapatite (ID nHAp) dopped with metal ions, to provide better mechanical support and eventually upgrade the biological activity of the new material. This composite hydrogel was prepared to create a new bone-like material for small bone defect repair using the tissue engineering approach. A simple synthetic method was applied to avoid complex synthetic procedures involved in bioconjugation [10]. Besides, the influence of two different pore forming methods on new porous material performances was investigated.

Composite HEMA/gelatin/PBAE 3D scaffolds with incorporated ion-doped nHAp and hybrid HEMA/gelatin/PBAE (without ion-doped nHAp) 3D scaffolds were fabricated in this study using two different pore-forming methods, cryogelation and porogenation. The scaffold structure, morphology, porosity, swelling ability, water contact angle, mechanical properties, and in vitro degradation were studied for the dependence on their composition and the pore-forming method. Biological tests were carried out to study the effect of the obtained materials on human mesenchymal stem cells cultured under osteogenic supplements.

## 2. Materials and Methods

### 2.1. Materials

HEMA, piperazine (PIPz, 99%), di(ethylene glycol)diacrylate and gelatin (type A, porcine), potassium persulfate (PPS, anhydrous), *N*,*N*,*N*′,*N*′-tetraethylethylene diamine (TEMED), *N*-Ethyl-*N*′-(3-dimethyl aminopropyl)carbodiimide hydrochloride (EDC), sodium hydrogen carbonate (NaHCO_3_), and polyethylene glycol sorbitan monolaurate (TWEEN 20) used in the cryogel and hydrogel synthesis were purchased from Sigma-Aldrich (St. Louis, MI, USA). All cryogel and hydrogel syntheses were performed in deionized water. Aqueous buffer solutions were prepared using potassium hydrogen phosphates (KH_2_PO_4_ and K_2_HPO_4_, Sigma-Aldrich) and deionized water. Materials used for the hydroxyapatite synthesis and doping were calcium nitrate pentahydrate (Ca(NO_3_)_2_ × 5H_2_O) (Sigma-Aldrich), magnesium nitrate hexahydrate (Mg(NO_3_)_2_ × 6H_2_O), strontium nitrate (Sr(NO_3_)_2_), gallium nitrate hydrate (Ga(NO_3_)_3_ × H_2_O), zinc nitrate hexahydrate (Zn(NO_3_)_2_ × 6H_2_O), and ammonium dihydrogen phosphate (NH_4_H_2_PO_4_) were obtained from Sigma-Aldrich, and urea ((NH_2_)_2_CO) was purchased from Alfa Aesar (Haverhill, MA, USA), and all were of analytical grade. All experiments were performed using lab-produced, ultra-distilled water.

### 2.2. Synthesis of Multidoped Hydroxyapatite

Sonochemical homogeneous precipitation method with thermally degraded urea was utilized to obtain the multidoped apatite [18]. HaP-precursor was created by mixing Ca- and dopants (Zn-, Sr-, Ga-, and Mg) precursors in an equivalent molar ratio (1:1:1:1). HaP precursor (2 wt%) was added to the mixture and preheated to 80 °C, followed by the addition of urea (12 wt%). Intensive sonification (with pulsation-to-relaxation periods on/off = 02:01 s, power *p* = 600 W, frequency f = 20 kHz, and amplitude A = 80%) was performed for 3 h, during which the slow thermal decomposition of urea resulted in precipitation. After sonification, the precipitate was aged in the supernatant for 15 h (at room temperature) followed by centrifugal separation (10 min at 6000 rpm) and drying under ambient conditions. Ultrasonic processor for high volume applications (VCX 750, Sonics & Materials, Inc., Newtown, CT, USA) was used for the synthesis.

PBAE synthesis was described in detail elsewhere [17]. The product was isolated pure and did not require any additional purification. 

#### 2.2.1. PHEMA/Gelatin Hydrogels Synthesis

**Figure 1:**
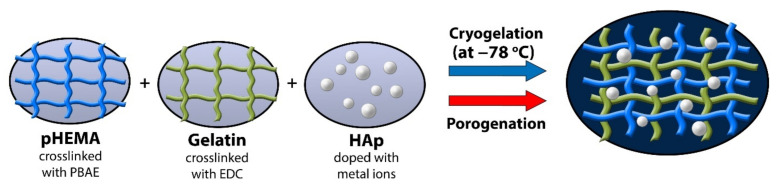
Hydrogel scaffolds synthetic route by cryogelation and porogenation.

PHEMA/gelatin hydrogels syntheses by cryogelation or porogenation are presented (Figure 1).

#### 2.2.2. Hydrogel Synthesis by Cryogelation

**Figure 2:**
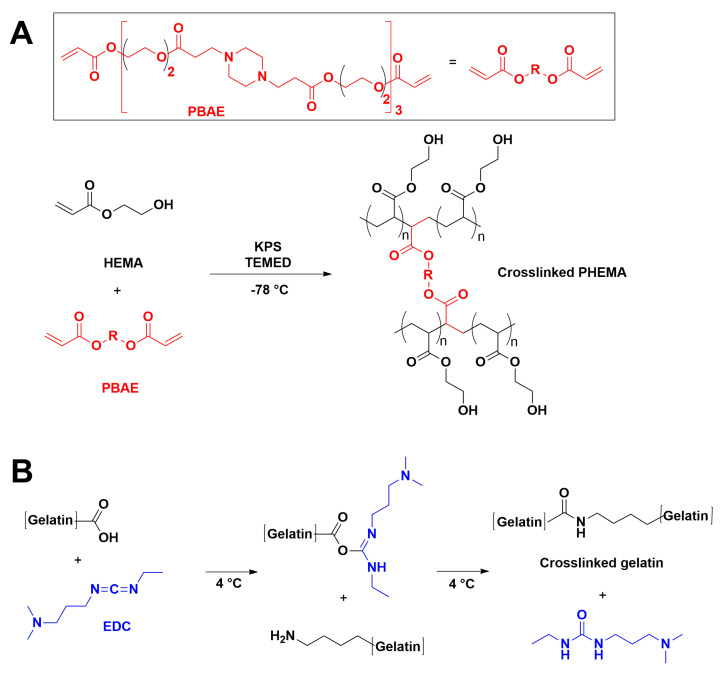
Crosslinking of HEMA with PBAE (**A**) and gelatin with EDC (**B**).

Cryogels with and without nHAp were synthesized using free-radical polymerization of HEMA at temperatures of −78 °C. Gelatin (307 mg), previously dissolved in deionized water (1 mL) by stirring at 40 °C, was added to a solution of HEMA (1.32 g, 10 mmol) and PBAE crosslinker *p* (0.13 g, 15% *w*/*w* with regard to the monomer) in deionized water (5 mL) that was stirring at r.t. This was followed by the addition of initiator (PPS, 150 mg) and activator (TEMED, 2 drops) to the reaction mixture, which was then transferred into a petri dish (5 cm wide, 0.6 cm height) and placed into a deep freezer (−78 °C) for 24 h. The formed cryogels were allowed to thaw at r.t. and submerged in a solution of EDC (1% solution in a mixture of acetone/deionized water (8/2)) at 4 °C overnight. Cryogel samples were washed with deionized water for 7 days to remove impurities. Water was changed daily. Swollen cryogels were frozen and freeze-dried. The cryogels were designated as in Table 1. Crosslinking of HEMA with PBAE and gelatin with EDC are presented in Figure 2.

#### 2.2.3. Hydrogel Synthesis by Porogenation

The same reaction mixture was polymerized in a glass vial, by heating the reaction mixture to 63 °C in a water bath, with continuous stirring for 5 min, with the addition of foaming agent NaHCO_3_ (54 mg) and TWEEN (4 mg) as a foam stabilizer. All synthesis details are presented in Table 1. After the polymerization was complete, the samples were cut into discs (7 mm diameter) that were washed with deionized water for 7 days to remove impurities. Water was changed daily. Swollen hydrogels were frozen and freeze-dried.

#### 2.2.4. Hydroxyapatite (HAp) Incorporation in Scaffolds

Mg, Sr, Zn, and Ga ion-doped HAp (synthesized as previously described by Kutjak et al.) [18] was incorporated during the polymerization process by adding 10% of the total hydrogel disk weight to the polymerization mixture. To achieve a homogenous distribution of HAp particles, continuous vigorous stirring was applied. Hydrogel scaffolds loaded with nHAp were marked as CH and PH, where CH stands for samples obtained by cryogelation and P for those obtained by porogenation. The samples without nHAp are designated as C and P.

### 2.3. Scaffold Characterization

#### 2.3.1. Fourier Transform Infrared Spectroscopy (FTIR)

Hydrogel composition was analyzed using FTIR spectra, recorded on a Thermo-Scientific Nicolet 6700 FT-IR diamond crystal spectrometer (Waltham, MA, USA), using the attenuated total reflectance (ATR) sampling technique. FTIR spectra were recorded over the wavelength range of 700–4000 cm^−1^.

#### 2.3.2. Scanning Electron Microscopy (SEM)

Morphological analysis of the scaffolds was performed with SEM (Jeol JSM-7600 F, Tokyo, Japan). Samples, that were previously freeze-dried (using Martin Christ-Alpha 1–2 LDplus, Osterode am Harz, Germany), were cut into slices, fixed on a holder using carbon tape, sputtered with gold (using BAL-TEC SCD 005, Wetter (Ruhr), Germany), and lyophilized in a vacuum chamber (VC 50 SalvisLab Vacucenter, Rotkreuz, Switzerland).

#### 2.3.3. Porosity Measurements

Solvent replacement procedure was used to calculate the hydrogel porosity, with glycerol (*ρ* = 1.2038 g/cm^3^) as a wetting medium. Dry hydrogel samples were immersed in glycerol for 24 h and weighed after removing excess glycerol from the sample:

(1) $$Porosity\;of\;hydrogel\;sample=\frac{\left(m_{g}-m_{i}\right)}{\rho V}\times 100$$

where *m_i_* is the starting weight of the dried hydrogel sample, *m_g_* is the weight of the hydrogel sample soaked with the wetting medium, *ρ* is the density of glycerol, and *V* is the volume of the hydrogel sample.

#### 2.3.4. Mechanical Characterization

A universal testing machine (Galdabini Quasar 50, Cardano Al Campo, Italy) was used to calculate the mechanical characteristics of the scaffolds by the application of a uniaxial compression with 100 N load cell at ambient conditions. The Young’s modulus (*E*) was quantified from the linear part of the stress/strain curve, with the average value of three measurements used for the final value.

#### 2.3.5. Swelling Study

The gravimetric method was used to calculate the equilibrium degree of swelling (*q_e_*) for all hydrogel samples. Dried hydrogel discs were submerged in phosphate buffer solution (pH 7.4 at 37 °C), imitating physiological conditions. Discs were taken out of the buffer solution at specific times, dried by removing excess water, and weighted. The equilibrium degree of swelling was determined using the formula:

(2) $$q_{e}=\frac{m_{s}-m_{i}}{m_{i}}$$

where *m_i_* is the initial weight of the dry gel and *m_s_* is the weight of the swollen sample, measured at selected time intervals, at the time of measuring. The equilibrium degree of swelling was plotted as a function of time.

#### 2.3.6. Water Contact Angle Measurement

The static water contact angle was measured using the sessile drop method by placing a drop (approximately 1 μL) of MilliQ water on the surface of the hydrogel. The measurements were performed using a Contact angle meter Theta Lite-Biolin Scientific (Phoenix, AZ, USA) (with measuring range 0–180 deg. and accuracy +/−0.1 deg., +/−0.01 mN/m) equipped with the camera with 640 × 480 resolution and a maximum measuring speed of 60 fps. All measurements were repeated at least four times for each hydrogel.

#### 2.3.7. In Vitro Degradation Study

In vitro hydrolytic degradation studies were carried out by immersing the hydrogel samples in a solution of phosphate buffer (pH 7.4 at 37 °C). Samples were removed from the buffer solution, dried at 40 °C until constant weight, and weighed every 2 weeks. Degradation was calculated as the remaining hydrogel mass percentage as a function of time, using the formula:

(3) $$Percent\;of\;remaining\;cryogel\;weight=\frac{m_{t}}{m_{i}}\times 100$$

where *m_i_* is the initial weight of the dry hydrogel and *m_t_* is the weight of the dried hydrogel sample at the time of measuring.

### 2.4. Biological Activity Studies

#### 2.4.1. Cell Expansion

Human mesenchymal stem cells were isolated from human bone marrow stromal aspirate (Lonza) as previously described [19]. The cells were used at passage three and cultured under standard cell culture conditions (37 °C, 5% CO_2_) in an expansion medium consisting of Dulbecco’s modified Eagle medium (DMEM, 41,966,029 Gibco, Thermo Fisher Scientific, Waltham, MA, USA), 10% fetal bovine serum (FBS, 15,240,062 Gibco, Thermo Fischer Scientific), 1% nonessential amino acids (NEAA, 111,140,035 Gibco, Thermo Fischer Scientific), 1 ng/mL basic fibroblastic growth factor (bFGF, PHG0369 Gibco, Thermo Fischer Scientific), and 1% antibiotic–antimycotic (Anti–Anti, 15,240,062 Gibco, Thermo Fisher Scientific). After 7 days, cells were trypsinized and 1 × 10^6^ cells were seeded on each scaffold by pipetting. After incubation for 90 min, 5 mL osteogenic media (DMEM, 10% FBS, 1% P/S/F, 50 μg/mL l-ascorbic acid (SIGMA), 100 nM dexamethasone (SIGMA), 10 mM beta-glycerolphosphate (SIGMA) was added. Constructs were cultured at 37 °C and 5% CO_2_ for up to 28 days, and culture media was changed every 2–3 days.

#### 2.4.2. DNA Quantification

The Quant-iT^TM^ PicoGreen^®^ dsDNA reagent kit (Invitrogen, Waltham, MA, USA) was used for DNA quantification. This allows for assessing the number of cells capable to adhere to the different scaffold types in comparison to adhering to tissue culture plastic (TCP). After 16 h of cell seeding, the scaffolds were carefully washed twice in phosphate buffer saline (PBS, Medicago, Quebec City, QC, Canada) to wash away nonadherent cells. The remaining cells were lysed in 0.2% (*v*/*v*) Triton X-100 and 5 mM MgCl_2_ solution using two steel beads and a Mini Beadbeater^TM^ (Biospec, Bartlesville, OK, USA) three times at 25,000 rpm for 10 s each time. Samples were placed on ice between cycles for cooling. Acellular scaffolds were used as negative controls. After 48 h incubation at room temperature and centrifugation, the Quant-iT^TM^ PicoGreen^®^ dsDNA reagent kit (Invitrogen) was used according to the manufacturer’s instructions. Fluorescence was read at an excitation wavelength of 480 nm and an emission wavelength of 520 nm with a plate reader (Tecan, Männedorf, Switzerland). The amount of DNA per sample was calculated according to the values of a DNA standard curve. DNA content of cells adhering to the scaffolds is presented relative to the DNA extracted from the same amount of cells seeded on TCP, which was set to 100%.

#### 2.4.3. Lactate Dehydrogenase (LDH) Activity

The release of LDH by cells, a stable cytoplasmic enzyme found in nearly all living cells, was used as an indicator of scaffold cytotoxicity. LDH activity was determined in the cell culture media after 48 h and 28 days of cell culture. The activity of the cytosolic enzyme was determined according to the manufacturer’s kit instructions (Roche Diagnostics, Rotkreuz, Switzerland). Results were presented relative to the LDH activity in the medium of cells cultured on TCP (0% cell death, low control) and of cells cultured on TCP and treated with 1% Triton X-100 (100% cell death, high control). To overcome intergroup scaffold background interference, absorbance obtained from each scaffold type without cells was previously subtracted from each corresponding group. Values are presented as mean ± SD.

#### 2.4.4. AlamarBlue Assay

AlamarBlue^®^ assay (Molecular Probes) was used as an indicator of cell metabolic activity. It was measured after 2, 7, and 28 days of cell culture on cell culture supernatant. Well plates containing the scaffolds were first washed once with PBS at 37 °C. The AlamarBlue assay was performed following the manufacturer’s instructions. Briefly, AlamarBlue solution was diluted 1:10 in control media and 700 µL of solution were added per well. Plates were then incubated for 1.16 h at 37 °C. For each sample, 100 µL of the sample was loaded into a 96-well black microtiter plate and fluorescence (excitation wavelength 535 nm, emission wavelength 595 nm) was measured with a plate reader (TECAN).

#### 2.4.5. RNA Isolation and Real-Time PCR Analysis

Trizol reagent (Invitrogen) was utilized to isolate total RNA following the manufacturer’s protocol. Each hydrogel sample was broken down with steel balls and a Minibead Beater (Biospec) at 25,000 rpm for 10 s per cycle (6 cycles). Hydrogel samples were kept on ice in between cycles. Total RNA (100 ng) was reverse transcribed to cDNA at 42 °C for 60 min with a High-Capacity RNA-to-cDNA kit (Applied Biosystems, Waltham, MA, USA) following the manufacturer’s protocol. The RNA was quantified using the Qubit 3.0 Fluorometer (Thermo Fisher Scientific) and the Qubit RNA HS Assay kit (Invitrogen).

Real-time PCR (Biorad CFX96) was achieved with TaqMan probe detection (Applied Biosystems). Real-time PCR was performed for glyceraldehyde-3-phosphate dehydrogenase (GAPDH, Hs02758991_g1), ACTB, Hs01060665_g1), collagen type I (COL1A2-I, Hs01028956_m1), alkaline phosphatase (ALPL, Hs01029144_m1), and osteocalcin (BGLAP, Hs01587814_g1). Each assay also contained a negative control without a cDNA template. qBase+ software was used for data analysis. Values were normalized to housekeeping genes (GAPDH and ACTB). Results are presented as fold change compared to sample P.

#### 2.4.6. Statistics

Data are presented as mean values ± SD. For cell experiments, a Kolmogorov–Smirnov test was performed to test normality. Differences between groups were assessed by one-way ANOVA with Bonferroni correction or Kruskal–Wallis test depending on their distribution. SPSS program for Windows (Chicago, IL, USA) version 17.0 was used. Results were considered statistically significant at *p* values < 0.05.

## 3. Results and Discussion

Nanocomposite hydrogels are organic–inorganic composite materials that have gained great interest in biomedical applications as artificial biomaterials for scaffolds. Their specific properties are achieved by the synergetic effect between organic hydrogels and inorganic nanoparticulate systems [20]. Nano-sized fillers are able to affect the physicochemical characteristics of the polymeric matrix, particularly the surface wettability and the mechanical properties, which are very important for further biological response [21,22]. On other hand, the polymeric matrix has the potential to control the release of nanofiller and their therapeutic effects [23]. Their advantages are found to be highly applicative in different spheres of biomedicine, including tissue engineering, diagnostic, and designing therapeutics [21,24]. In this work, new nanocomposite hydrogels were synthesized using a HEMA/gelatin/PBAE hydrogel combined with nHAp dopped with different ions (ID nHAp). Porous three-dimensional IPN hydrogels were obtained by cryogelation or porogenation. To assess the impact of doped nHAp on the scaffold properties in each set, one sample was made with ID nHAp and the other without it. The cryogels samples were marked as CH and C, and the samples obtained using porogen as PH and P, where H stands for nHAp.

### 3.1. Structural Characteristics of Scaffolds

**Figure 3:**
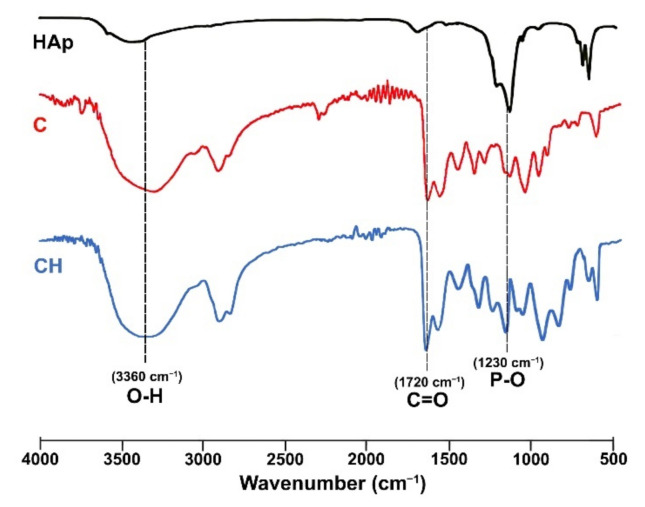
FTIR spectra of HAp and C and CH cryogels.

The chemical structure characteristics of all samples (C, CH, P, and PH) based on HEMA, gelatin, and PBAE, with and without doped nHAp, were examined using Fourier transform infrared (FTIR) spectroscopy to examine polymer–particle interaction. Figure 3 shows the FTIR spectra of nHAp and the cryogels with and without nHAp (Figure 3), as well as the doped nHAp spectrum. The nHAp spectrum shows a peak at 960 cm^−1^ for the asymmetric stretching vibration of PO_4_^3−^, and strong bands at 1230 and 1026 cm^−1^ for P–O [25]. The bands present at 607 cm^−1^, 563 cm^−1^, and 609 cm^−1^ are due to the bending mode of P–O bonds in the phosphate group [25]. Observable bands in the C cryogel spectra are 3360 cm^−1^ for O–H and N–H stretching, 2944 cm^−1^ for C–H stretching of alkyl groups, 1720 cm^−1^ for C=O stretching of ester carbonyl group, 1640 cm^−1^ for C=O stretching of the amide group (amide I), and 1549 cm^−1^ for N–H bending. A change of the intensity of the peaks at 3360, 1720, and 1230 cm^−1^ confirmed the presence of nHAp incorporated in the scaffolds.

### 3.2. Scaffold Morphology

The morphology of the scaffolds plays an important role in bone tissue regeneration. Highly porous scaffolds with interconnected pores are needed to obtain a homogeneous cell distribution throughout the scaffold, as well as the efficient diffusion of nutrients, oxygen, and waste materials, especially in the absence of a functional vascular system. Therefore, it is essential for cell survival, growth, and vascularization. Adequate porosity and interconnectivity are also required to facilitate cell migration within the scaffold, such that cell growth is enabled in the right direction. The pore sizes are also very important. Large pores, around 100 μm or more, are needed to direct the tissue formation and function in the scaffolds due to the higher amount of functional units necessary for the regeneration of various tissues, including bone [26].

**Figure 4:**
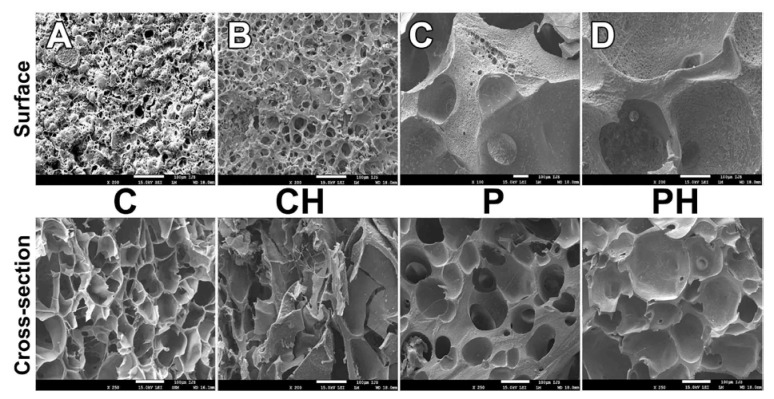
Representative SEM micrographs of the surface and a cross-section for each group (**A**) C, (**B**) CH, (**C**) P, and (**D**) PH (scale bar: 100 μm).

Morphological characteristics of the scaffolds were studied using SEM. SEM micrographs of the cross-section (Figure 4) for the cryogels (C, CH) show highly porous structures with interconnected, irregular elongated pores, where small pores are surrounded by polymeric thick walls of macropores. Pores with a diameter of about 100 μm and larger were detected, which confirms that this material microstructure is suitable for TE purposes. Samples obtained using porogen (P, PH) also have a highly porous structure with large, but less interconnected pores. The pore shape for P and PH is different from that for the C and CH samples, as they predominantly have a round shape, probably due to the presence of a foam stabilizer during pore formation. The cryogels also have higher porosity (table) than the samples obtained by porogenation. As can be seen, the SEM micrographs for CH have more irregular pores than the C scaffold. This is probably because the nHAp particles in the polymer solution perturbed the solvent crystallization to some extent. The micrographs for the PH samples also differ from those for the P samples, showing more irregular pores with a greater contribution of large pores. There is no evidence of the phase separation or agglomeration of the nHAp particles, meaning that the scaffold components are compatible and that the nanoparticles are distributed homogeneously in the material. The SEM images of the surface are different. There is also a highly porous structure with interconnected pores, but higher porosity is obtained for the P and PH samples due to the different pore-forming processes, and probably also due to the solvent effects in the C and CH samples during the cryogelation process.

### 3.3. Scaffold Porosity

The porosity of scaffolds was determined by the solvent replacement method using glycerol as a wetting medium. As could be expected, the addition of ID nHAp in both types of samples led to slightly lower porosity, as can be seen from Table 2. For the cryogels, the porosity was determined to be 83.92% for C and 78.96% for CH, while for the samples obtained using porogen, the porosity decreased from 71.17% for the P sample to 65.66% for the PH sample.

### 3.4. Scaffold Swelling Properties

**Figure 5:**
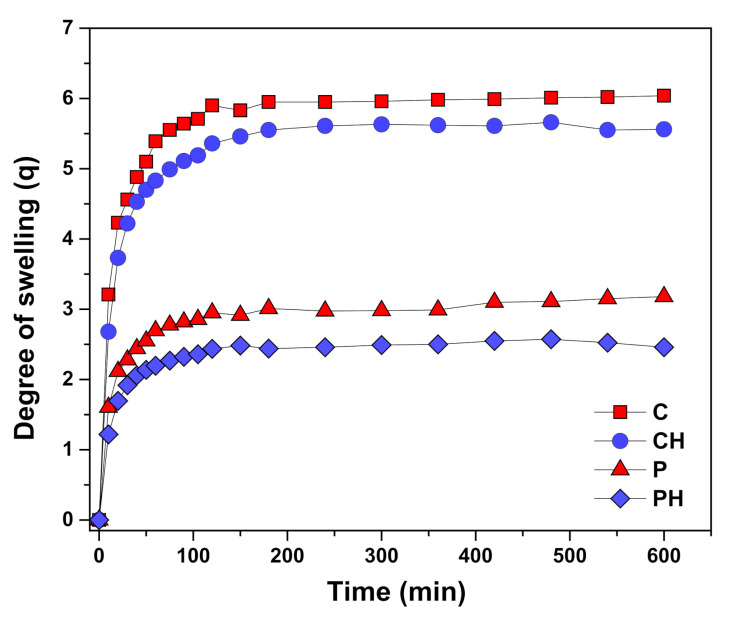
In vitro swelling profiles of the designed scaffolds.

In vitro swelling studies were performed in a pH 7.4 buffer at 37 °C to mimic the physiological conditions. The results obtained for the scaffold swelling are presented in Figure 5 as the degree of swelling (*q*) values versus time. The fast-swelling behavior [27], typical for highly hydrophilic and porous hydrogel materials, can be observed for all samples from a very steep slope of the swelling curves at the beginning of the swelling process. In general, the scaffolds obtained by cryogelation (CH and C) swell considerably more than those obtained by porogenation (PH and P). The equilibrium degree of swelling (*q_e_*) values for cryogels were 5.12 ± 0.05, and about 5% lower values of 4.87 ± 0.04 were obtained for composite cryogels. Lower *q_e_* values were obtained for the samples in which porogen was used, with 1.93 ± 0.03 for the samples without nHAp and 1.74 ± 0.03 (about 10% lower) for the composite samples. It is obvious that the addition of inorganic Hap slightly lowers the degree of swelling depending on the pore forming method, which agrees well with the porosity results.

### 3.5. Hydrophilicity of Scaffolds

Surface wettability is a significant characteristic of a material used in biomedical applications [28]. Wettability is the tendency of a liquid to spread on a solid substrate and is generally measured by measuring the contact angle, i.e., the angle between the substrate surface and the tangential line drawn at the triple point of the three phases (solid, liquid, and gas). The origin of the macroscopic water wetting/nonwetting phenomenon at polymer surfaces should ultimately be explainable in terms of the interaction between the atoms of the water molecule and the surface of the polymer. The wettability depends on porosity, surface roughness, and chemical composition.

**Figure 6:**
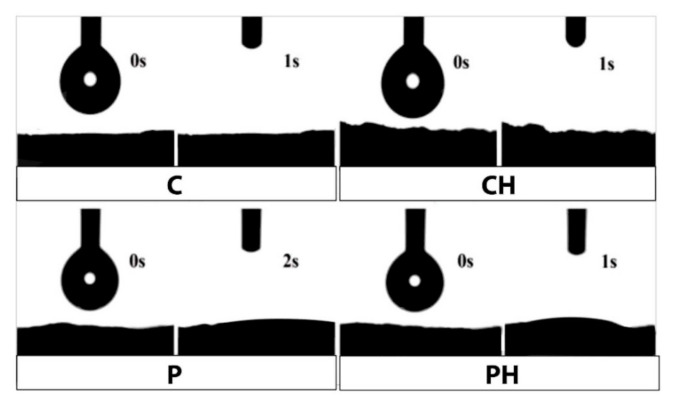
Hydrophilicity of C, CH, P, and PH hydrogel scaffolds.

The determination of the wettability is based on the measurement of the material surface water contact angle. Contact angle measurements revealed that the scaffold surfaces are highly hydrophilic (Figure 6). Immediately after touching the surface, the water drop is completely wetting the surface and disappearing. This behavior was expected due to the high content of hydrophilic groups in the PHEMA/gelatin/PBAE (with and without ID HAp) scaffold surface, allowing for a high wettability which is very important for successful cell adhesion, proliferation, and differentiation [29,30].

### 3.6. In Vitro Degradation Behavior

One of the most essential parameters to evaluate the suitability of biomaterials for use as scaffolds and implants is the biodegradability. Degradation will provide new space for the tissue to grow in and induce regeneration, so it is of utmost importance to synchronize the rate of degradation with the rate of growth of new tissue. Synthetic hydrogels offer the advantage of tailorable degradation, varying with chemical composition and the ratio of its component materials, among many other parameters. The physicochemical characteristics of the gelatin/HEMA/PBAE hydrogel scaffolds could be fine-tuned using an adequate gelatin/HEMA/PBAE ratio and the PBAE crosslinker structure, as determined in our previous work [17]. For the scaffolds used herein, we selected the most suitable gelatin/HEMA/PBAE ratio for the cryogel and the porogenated scaffold synthesis. These two scaffold groups showed different degradation behavior over 16 weeks (Table 2).

The mass loss percent after 16 weeks for the cryogel samples was 23.17 ± 0.85% for the samples without nHAp and 21.93 ± 1.12% for those containing nHAp. Scaffolds obtained by porogenation had lower mass loss values for the same period, 9.24 ± 0.67% and 7.55 ± 0.69%, where higher values are for the scaffolds with nHAp. As can be seen, the difference in the degradation rate is pronounced for the different pore forming methods. Cryogelation leads to faster water absorption by the interconnected pores, which contributes to a higher rate of hydrolytic degradation. Incorporating nHAp showed a slight decrease in the degradation for both types of pore-forming methods. This result shows the important impact of the method of pore formation on the final scaffold properties.

### 3.7. Scaffold Mechanical Properties 

The elastic modulus of the scaffolds is a critical physical property because it is one of the important factors which determines their use in biomedical applications. Ideally, it should match the mechanical properties of the physiological tissue surroundings where the scaffold is implanted, be strong enough to provide the structural support for cell attachment, as well as succeeding tissue growth until the new tissue is formed. In that context, designing scaffolds using a polymeric matrix with embedded inorganic nanoparticles is useful tool for controlling the mechanical strength of soft gels [31].

For the scaffolds developed herein, the composite scaffolds had higher Young’s modulus values of 10.14 MPa for the porogenated samples (PH) and 5.87 MPa for the cryogels (CH) than the samples without the nHAp component, 8.63 MPa (P) and 4.25 MPa (C), results which validate the better mechanical properties of the composite systems for both types of scaffolds. The porogenation technique resulted in higher modulus values than in the case of cryogels due to the lower porosity (Table 2). It can also be seen that the values for Young’s modulus are following the results obtained for the porosity, the degree of swelling, and the degradation rate (Table 2). The elongation at the break for the composite scaffold values were 24.37% for CH and 17.58% for PH, which is slightly lower than for the samples without nHAp, namely 26.96% for C and 20.45% for P. It can be seen that the mechanical properties of the HEMA/gelatin/PBAE polymer scaffolds were improved by the nHAp addition. The method of pore formation also has a considerable influence on hydrogel mechanical properties. Integrating nHAp manifested as an increase of strength and a slight decrease in elongation at the break for both types of samples.

### 3.8. Scaffold Biological Properties

The PHEMA/gelatin hydrogels were assessed in vitro for their potential use as 3D scaffolds for bone regeneration. Although different methodologies are being applied in TE, cell implantation, as one of the most frequently used, was also applied here. Living cells were seeded into a scaffold. Successful tissue regeneration is achieved when the scaffolds finally degrade in an appropriate time scale, leaving behind only the healthy/regenerated tissue [2,32,33]. Such scaffolds should be nontoxic and support cell adhesion, proliferation, and osteogenic cell differentiation. To assess the biological properties of the developed materials, the scaffolds were seeded with osteoblast progenitors human bone marrow-derived stromal cells (hBMSCs). The performance of the scaffold materials was compared to cells cultured on 2D TCP surfaces.

### 3.9. Cell Adhesion

**Figure 7:**
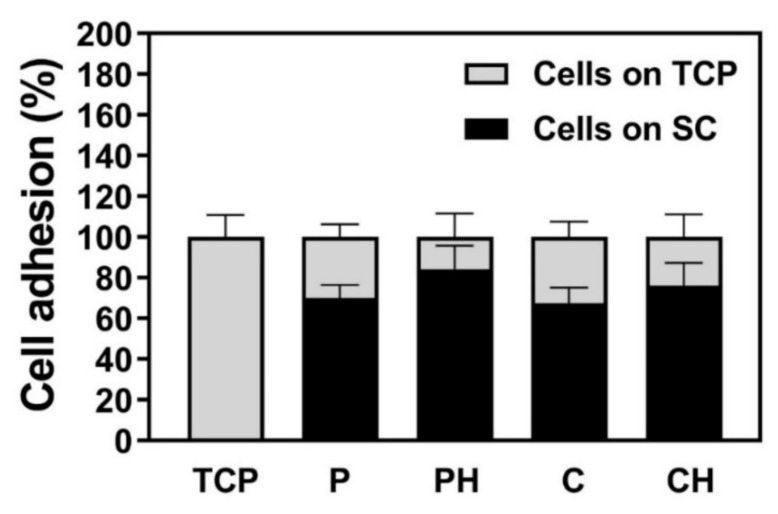
Cell adhesion measured as the amount of DNA from cells adhering to tissue culture plastic (TCP, grey) or the scaffold (black) 16 h after cell seeding. Data represent the mean ± SEM (*n* = 4).

A DNA assay was performed to assess the number of cells able to adhere to the different scaffold types. The negatively charged (air or oxygen) and hydrophilic surface of TCP can increase the nonspecific adsorption of cell media constituents and enable subsequent coatings that can further promote cell adhesion. More specifically, we evaluated whether the presence of HAp nanoparticles in the scaffold material (HG-based or PLLA Dox-based) and/or the scaffold fabrication method (porogen, cryogel) influenced the amount of cells able to adhere to the scaffolds. Both the cells adhering to the scaffold and those adhering to the TCP were evaluated, the sum of which represents all adherent cells (100%). A high cell adhesion rate of >60% was observed on all scaffold types studied (Figure 7). This could, for example, be thanks to the hydrophilicity of the scaffolds, which could favor protein absorption and mediate the cell–material interaction. Besides, gelatin, obtained by the partial hydrolysis of native collagen, can ensure numerous functional groups appropriate for strong interactions with a large variety of cells [34,35]. Variations on the topography due to HAp doping might cause differences in the distribution of adhesion proteins [36], increasing the cell adhesion in Hap-doped materials compared to its nondoped material, although the results were not statistically different. No significant differences were observed between the porogen and the cryogel, either doped or nondoped HAp.

### 3.10. Influence of Scaffolds on Cell Viability

**Figure 8:**
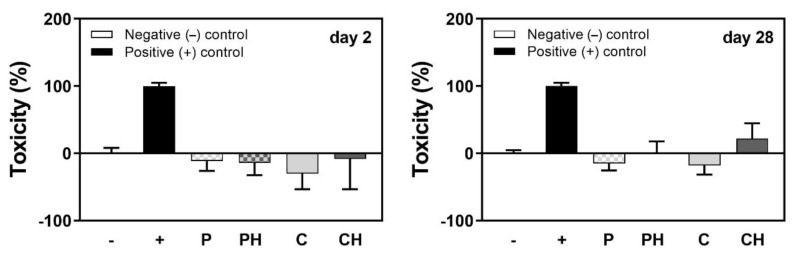
Toxicity represented as LDH activity relative to cells treated with Triton X-100 (+ control, representing 100% cell death) and cells cultivated in TCP (− control, 0% cell death) measured in the culture media after 2 or 28 days of cell culture on either P, PH, C, or CH scaffolds.

LDH activity measurements were performed to measure the cytotoxicity of the different scaffold types as an indirect measure of cell viability after 2 and 28 days of cell culture. All scaffold types can be considered safe (toxicity occurs in ≤50% of the cells) [37] for the cells after both short (2 days) and longer-term (28 days) cell cultures (Figure 8). In addition, no significant differences were observed in the cell toxicity among the groups, indicating no toxicity of the scaffold materials for the cells, but ensuring cell viability.

### 3.11. Cell Metabolic Activity

**Figure 9:**
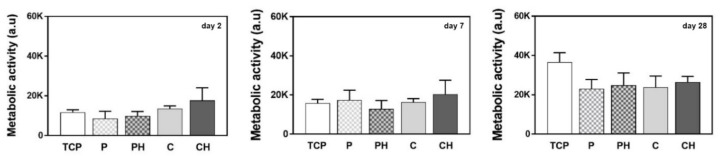
Determination of the cell metabolic activity after culturing cells for 2, 7, and 28 days in P, PH, C, or CH scaffolds.

AlamarBlue is a commonly used assay to determine metabolically active cells, giving insight into cell viability, and cell proliferation [38,39]. After 2 and 7 days of cell culture, all the scaffold types equally supported viability (Figure 9). The cell metabolic activity in the CH was 30% and 25% higher after 2 and 7 days of culture, respectively, compared to C, although not statistically different. Interestingly, after 28 days of culture, cell metabolic activity showed the highest increase for the TCP reference group, although this difference was not statistically significant. Overall, these data indicate that the cell metabolic activity is similar to TCP on each of the scaffold materials used.

### 3.12. Upregulation of Osteogenesis-Related mRNA Levels

**Figure 10:**
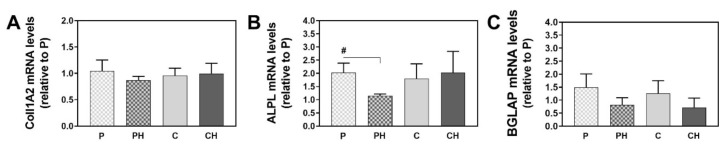
Gene expression levels of COLL1A2 (**A**), ALPL (**B**), and BGLAP (**C**) after 28 days of culture of hBMSCs on the different scaffold materials. Values were normalized to a housekeeping gene (GAPDH and ACTB) and expressed as fold change relative to P, which was set to 1. Values represent the mean ± SD (n = 3) ^#^ *p* < 0.05.

Osteogenesis-related genes collagen type 1 (Coll1A2, bone ECM protein), alkaline phosphatase (ALPL, an early marker of osteogenic differentiation), and osteocalcin (BGLAP, a late marker of osteogenic differentiation) were evaluated after 28 days of culture (Figure 10). For the porogenated scaffolds, contrary to what was expected, the integration of nHAp seemed to reduce the expression of COLL1A2 and BGLAP and significantly downregulated at 0.5 fold change the ALPL mRNA levels compared to the scaffolds without nHAp. For the cryogels, this trend was less pronounced. Overall, it seems as if osteogenic differentiation is slower or less pronounced when nHAp was integrated into the gels.

## 4. Conclusions

New composite HEMA/gelatin/PBAE 3D scaffolds with incorporated ID nHAp and hybrid HEMA/gelatin/PBAE 3D scaffolds without ID nHAp were successfully fabricated using cryogelation (CH, C) and porogenation (PH, P) pore forming methods. The physicochemical characterization of the scaffolds revealed that the incorporation of nHAp led to changes of the structure, morphology, porosity, swelling ability, water contact angle, mechanical properties, and in vitro degradation, as well as in vitro biological properties in CH and PH scaffolds, relative to C and P. The pore forming method affected the scaffold characteristics to an even greater extent than the incorporation of nHAp. The composite samples had considerable elasticity and a rather high mechanical strength, but slightly lower degrees of swelling, percent of mass loss after 16 weeks, and porosity values. Furthermore, the scaffold fabrication method and the inclusion of nHAp influenced very little the cell adhesion capacity and toxicity. A high cell adhesion rate of >60% was observed on all the scaffold types studied, and the scaffold materials were proven to be biocompatible and nontoxic. According to the cell metabolic activity after 2 and 7 days of cell culture, all scaffold types equally supported viability. Cell metabolic activity after 2 and 7 days of culture was 30% and 25% higher in the CH compared to C, although not statistically different. After 28 days of culture, cell metabolic activity showed the highest increase for the TCP reference group, although this difference was not statistically significant. After 28 days of culture, cell metabolic activity indicates that the cell metabolic activity is similar to TCP on each of the scaffold materials used. Osteogenic differentiation was slower or less pronounced when nHAp was integrated. The combination of hydrogels with inorganic nHAp in the PHEMA/gelatin/PBAE scaffolds resulted in superior properties for applications in the biomedical field, probably owing to synergetic effects, which lead to improvements in the mechanical behavior and the interactions with biological species.

## Figures and Tables

**Table 1:** The composition and designation of the hydrogel samples.

Sample	Component 1	Component 2	Cross-Linker for pHEMA	Cross-Linker for Gelatin	Initiator/Initiation Catalyst	Pore Formation Method (Foaming Agent/Stabilizer)	nHAp Doped
C	HEMA	Gelatin	PBEA	EDC	APS/TEMED	Cryogelation	-
CH	HEMA	Gelatin	PBEA	EDC	APS/TEMED	Cryogelation	yes
P	HEMA	Gelatin	PBEA	EDC	APS/TEMED	Porogenation (NaHCO_3_/TWEEN)	-
PH	HEMA	Gelatin	PBEA	EDC	APS/TEMED	Porogenation (NaHCO_3_/TWEEN)	yes

**Table 2:** Equilibrium degree of swelling, Young’s modulus, porosity, percentage of degradation, and elongation at break percentage for each group.

Sample	Equilibrium Degree of Swelling	Young’s Modulus (MPa)	Porosity (%)	Percentage of Mass Loss (after 16 Weeks)	Elongation at Break (%)
C	5.12 ± 0.05	4.25 ± 0.22	83.92	23.17 ± 0.85	26.96 ± 1.12
CH	4.87 ± 0.04	5.87 ± 0.24	78.96	21.93 ± 1.12	24.37 ± 1.18
P	1.93 ± 0.03	8.63 ± 0.28	71.17	9.24 ± 0.67	20.45 ± 1.36
PH	1.74 ± 0.03	10.14 ± 0.31	65.66	7.55 ± 0.69	17.58 ± 1.41

## Data Availability

Not applicable.
